# An Integrated UAV Trajectory Adaptation Framework for 5G Highway Vehicular Communications

**DOI:** 10.3390/s26165173

**Published:** 2026-08-15

**Authors:** Ignacio Vidal, Sandy Bolufé, Karel Toledo

**Affiliations:** Department of Electrical Engineering, University of Santiago of Chile, Santiago 9170124, Chile; ignacio.vidal.c@usach.cl (I.V.); sandy.bolufe@usach.cl (S.B.)

**Keywords:** 5G communication, path planning, throughput, unmanned aerial vehicles, vehicular networks

## Abstract

This paper investigates the use of unmanned aerial vehicles (UAVs) as flying base stations (BSs) to enhance fifth generation (5G) vehicular communications on highways, where traffic congestion and fluctuating user demand can challenge the capacity of terrestrial infrastructure. While UAV-assisted vehicular networking has attracted significant attention, many existing studies rely on simplified mobility, propagation, or communication models that limit the assessment of practical deployment performance. To address these limitations, we develop a realistic UAV-assisted vehicular networking framework that integrates microscopic traffic simulation through Simulation of Urban MObility (SUMO), network control via Traffic Control Interface (TraCI), and standard-compliant 5G communication modeling using MATLAB R2025b 5G Toolbox. The framework incorporates a 3rd Generation Partnership Project (3GPP) rural macro cell (RMa) highway scenario, detailed clustered delay line (CDL)-based channel characterization, and cross-layer communication procedures. Within this framework, we propose a low-complexity trajectory optimization strategy that adapts the UAV position in real time to maximize the average received signal to noise ratio (SNR) while respecting practical motion constraints. Simulation results demonstrate that adaptive UAV positioning enhances communication performance, achieving mean SNR gains of up to 2.04 dB, throughput improvement of up to 11.2%, and block error rate (BLER) reductions of up to 27.3%. These findings highlight the potential of UAV-assisted communications to enhance user-perceived quality of service (QoS) for bandwidth-demanding vehicular applications under realistic 5G highway operating conditions.

## 1. Introduction

Modern connectivity increasingly relies on seamless interaction between people, devices, and their environments, enabled by advances in wired and wireless technologies. Vehicular networks have become a key research area due to their potential to enhance road safety, traffic efficiency, and user experience [1]. As autonomous driving advances, vehicular networks must support large-scale, real-time data exchange for emerging cooperative and intelligent transportation services. This increasing demand exposes the limitations of conventional vehicular networks [2].

Vehicular networks often experience data transmission bottlenecks during heavy traffic and peak hours, when high message volumes strain terrestrial infrastructure and degrade signal reception and processing. These limitations become more severe during unexpected events or disasters that can disrupt ground communication systems. In such conditions, UAVs offer a practical complement to vehicular networks by providing flexible and wide-area coverage, enhancing system resilience, and supporting secure and efficient communication [3].

The incorporation of UAVs into vehicular networks is envisioned, primarily due to their capacity to improve connectivity in remote and underserved areas where traditional infrastructure is unavailable or costly to deploy [4]. Their high mobility enables quick deployment in critical situations, such as natural disasters or large-scale accidents, improving QoS by maintaining reliable communication links and supporting optimized rescue operations. Furthermore, their adaptability makes them ideal for temporary events, such as concerts or sporting events, where short-term communication infrastructure is needed, as well as for specialized applications like aerial mapping, traffic monitoring, and real-time data collection [5]. As a result, research and development efforts in vehicular networks are intensifying, focusing on leveraging UAVs to improve network reliability, expand coverage, and boost overall system performance.

A relevant application scenario involves deploying UAVs as flying BSs or roadside units (RSUs) to support vehicles on highways. UAV-assisted vehicular communication in highway environments has gained substantial attention due to its potential to enhance coverage and reliability. For example, ref. [6] introduces an integrated aerial-ground architecture that enables UAV-vehicle collaboration through a combined vehicle-to-UAV (V2U) and vehicle-to-vehicle (V2V) framework for diverse highway applications. Similarly, ref. [7] investigates UAVs as aerial relays to strengthen or restore weak communication links, reducing latency and improving overall connectivity. UAVs have also been shown to effectively complement terrestrial infrastructure by increasing throughput and ensuring QoS for in-vehicle services such as on-demand streaming [8].

**Figure 1:**
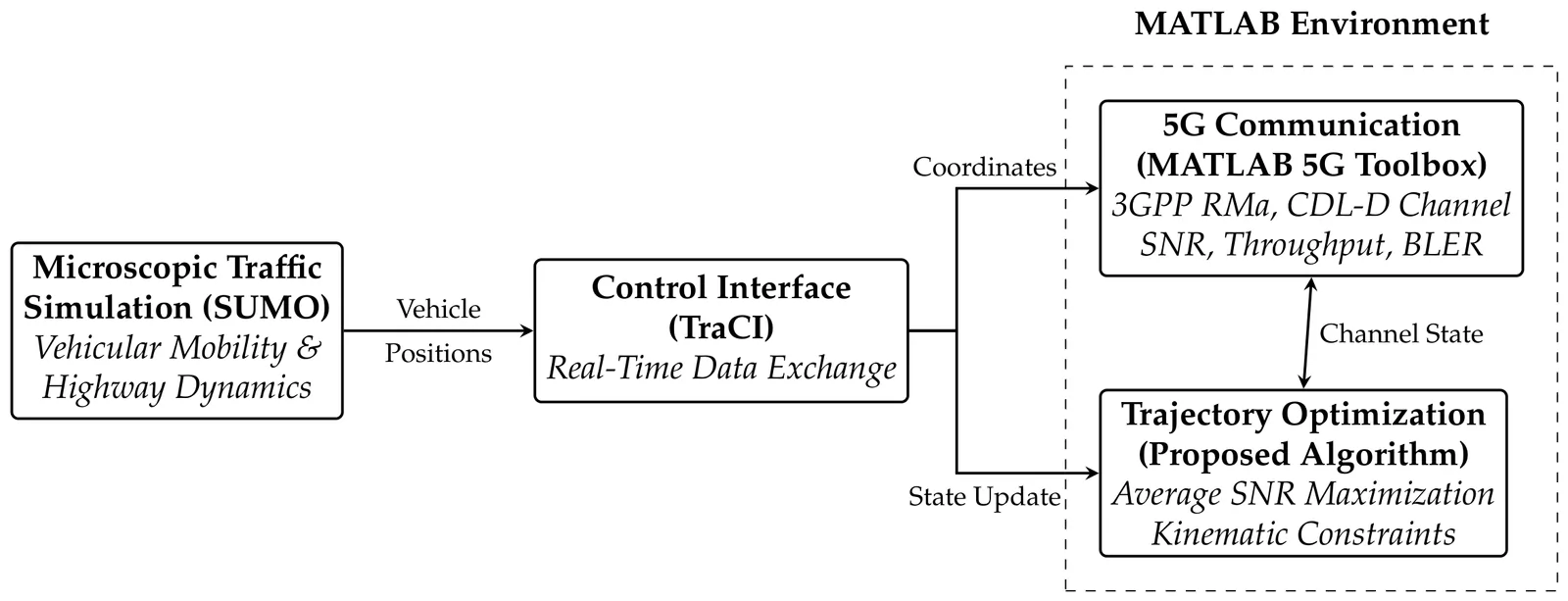
Schematic of the integrated UAV-assisted vehicular networking framework, illustrating the interaction between mobility simulation, network control, and 5G trajectory optimization.

To meet QoS requirements, appropriate UAV route planning must be carried out to increase the SNR at each vehicle, which in turn enhances the successful data received and reduces the BLER. Then, the primary objective is to optimize the trajectory of a UAV in vehicular networks at each time step to maximize the average SNR at the receiver side, thereby guaranteeing a given QoS. A feasible solution to this problem is addressed by a suboptimal greedy algorithm that finds the next spatial coordinate of the UAV. To guarantee deployment feasibility, it is imperative to examine and assess realistic conditions, which are approached from three directions: (i) microscopic simulation of vehicle movement explicitly using SUMO [9], (ii) integration of a UAV into the vehicular network employing TraCI, and (iii) 5G communication links and a standard-compliant propagation channel provided by MATLAB 5G Toolbox. Figure 1 presents the overall architecture of the proposed framework, illustrating the information flow and interaction among the microscopic traffic simulation, TraCI interface, 5G communication model, and UAV trajectory optimization modules. This strategy positions the work within a realistic and cohesive framework that integrates mobility dynamics, 5G communication procedures, and channel modeling, thereby enabling a comprehensive evaluation of the proposed trajectory optimization approach under conditions that closely approximate real-world UAV-aided vehicular networks.

Our main contributions can be summarized as follows:

- We develop a realistic UAV-assisted vehicular networking framework for highway environments by integrating microscopic traffic simulation through SUMO, network control via TraCI, and standard-compliant 5G communication modeling using MATLAB 5G Toolbox. The framework considers a 3GPP RMa deployment scenario and detailed vehicular mobility dynamics.
- We propose a low-complexity trajectory optimization strategy for UAV-assisted communications that maximizes the average received SNR while respecting practical UAV motion constraints, including discrete movement, velocity limits, and acceleration restrictions. The resulting approach is suitable for real-time deployment and online decision-making.
- We incorporate realistic wireless channel characterization based on 3GPP CDL models, together with medium access control (MAC)-layer scheduling and hybrid automatic repeat request (HARQ) procedures, enabling a cross-layer evaluation of communication performance beyond conventional path loss-based analyses.
- We conduct a comprehensive performance evaluation under realistic highway traffic conditions, demonstrating the impact of adaptive UAV positioning on key communication metrics, including SNR, throughput, and BLER for bandwidth-demanding vehicular applications.

The remainder of the paper is organized as follows. Section 2 reviews the literature on UAV-assisted vehicular networks. Section 3 outlines the system model, including mobility, architecture, channel, and metrics. Section 4 formulates the UAV trajectory optimization problem and presents the proposed algorithm. Section 5 provides simulation parameters and performance evaluation. Section 6 concludes the paper.

## 2. Related Work

The integration of UAVs into vehicular networks offers significant advantages, including enhanced coverage, optimized resource allocation, and support for advanced vehicular applications. This section reviews state-of-the-art research on UAV-assisted vehicular networks, categorized by their primary functional objectives, and contrasts them with the proposed framework to address the limitations in modeling fidelity and real-time optimization.

A primary application of UAVs is extending coverage and ensuring connectivity where RSUs are unavailable or obstructed. Su et al. [10] explored single- and multi-UAV relaying to mitigate RSU malfunctions, ensuring high-throughput links for the Internet of Vehicles (IoV). Similarly, Li et al. [11] introduced a two-way relaying system for millimeter-wave networks, jointly optimizing relay selection and scheduling to meet throughput demands. Optimization of relay trajectories is critical for these systems. He et al. [12] approached relay selection as a multi-objective optimization problem considering energy and delay, while their subsequent work [13] utilized Newton’s method to optimize trajectories for delay-sensitive secure transmission. Further addressing physical-layer issues, Sun et al. [14] investigated uplink power allocation in UAV-aided platooning non-orthogonal multiple access (NOMA) systems, employing a power-adjusting mechanism to maintain signal to interference plus noise ratio (SINR) as vehicles diverge from the platoon.

As network complexity increases, stringent security measures and efficient routing protocols become imperative. Khan et al. [15] proposed an artificial gorilla troop optimization strategy to enhance trust analysis and packet delivery in ground-to-UAV communication. To combat urban obstructions, Oubbati et al. [16] developed a flooding-based routing solution where vehicles and UAVs cooperate to recover from path failures. Regarding resource management, Zhao et al. [17] focused on minimizing energy consumption in integrated platooning networks. Ismail et al. [18] proposed a cooperative protocol for channel resource allocation based on line of sight (LOS) probability, enhancing network throughput.

Recent research has shifted toward mobile edge computing (MEC) and real-time data processing. Sun et al. [19] presented a UAV-enabled MEC system that offloads IoV application data to UAV-mounted servers, optimizing task sequencing to minimize response time. Similarly, Samir et al. [20] focused on trajectory optimization to maintain the age of information (AoI) for time-sensitive sensor data streams. In the domain of traffic management, UAVs serve as effective aerial sensors. Jobaer et al. [21] integrated UAVs with ad hoc routing to share real-time congestion data in urban environments. For highway scenarios, recent studies have introduced probabilistic trajectory planning for traffic density estimation [22], automated inspection schemes [23], and distributed WSN-assisted surveillance frameworks [24]. Other works have addressed specific challenges such as energy-aware 3D deployment in complex interchanges [25] and clustering optimization [26]. A taxonomy of these approaches is summarized in Table 1, highlighting their primary objectives and key characteristics or limitations.

While numerous studies have investigated UAV-assisted vehicular networks, they often focus on theoretical analyses or simplified performance metrics that fail to capture the concurrent complexities of 5G propagation, microscopic mobility, and UAV physical constraints. This work addresses these gaps by diverging from the continuous-space iterative solvers [13] or probabilistic planning frameworks [22] found in the literature, proposing instead a discrete-motion greedy algorithm that respects actual kinematic limits, specifically velocity and acceleration, to ensure low-latency and real-time decision-making. Furthermore, we replace common log-distance or free-space path loss assumptions with 3GPP-compliant RMa and CDL models to accurately evaluate standard-compliant 5G mmWave channels. By utilizing SUMO and TraCI to model microscopic vehicular interactions rather than macroscopic flows, the UAV trajectory is designed to adapt to instantaneous traffic behavior. This cross-layer approach, which incorporates MAC-layer scheduling and HARQ mechanisms, shifts the focus from theoretical capacity bounds toward a realistic maximization of user-perceived QoS within a cohesive simulation framework.

## 3. UAV-Aided Vehicular Network

**Figure 2:**
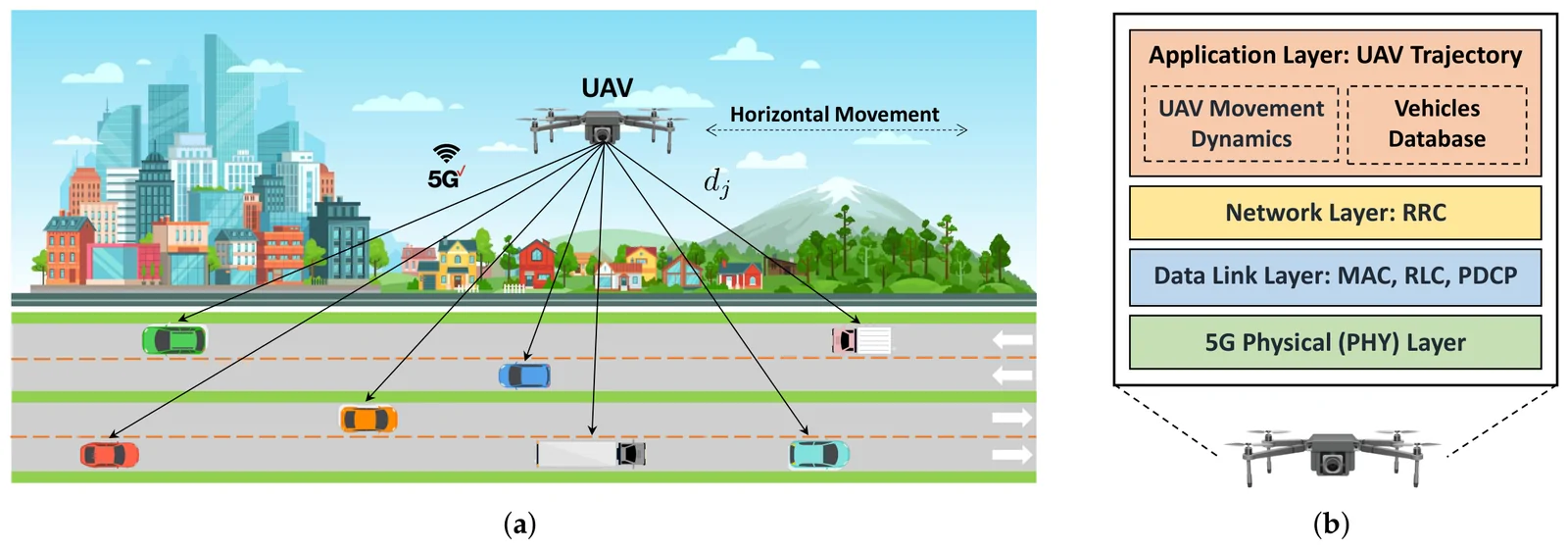
System model of UAV-assisted vehicular network. (**a**) Highway scenario. (**b**) Layer model of the UAV.

The wireless network consists of a UAV acting as a flying RSU, traversing a highway of length *s* and providing coverage to a set of *N* vehicles moving along the road, as illustrated in Figure 2a. The vehicles behave as mobile entities whose positions evolve continuously within the UAV’s coverage area, enabling a realistic evaluation of communication performance and QoS optimization in dynamic highway environments. The UAV operates according to the layer model shown in Figure 2b. For simplicity, time is discretized to describe the UAV movement pattern where at each time slot, the UAV may either remain at its current position or relocate to one of a finite set of reachable surrounding positions. Deployed at height $z_{u}$, the UAV moves in a two-dimensional (2D) Cartesian plane.

The vector $p_{u}\left(t\right)=\left[x_{u}\left(t\right),y_{u}\left(t\right),z_{u}\right]$ represents the UAV’s position at each time slot *t*, while the vector $p_{v}\left(t\right)=\left[x_{v}\left(t\right),y_{v}\left(t\right),0\right]$ defines the trajectory of a vehicle. The distance $d_{j}\left(t\right)$ between the UAV and the *j*-th vehicle over the entire simulation time *T* is determined by the Euclidean norm ∥·∥ and is given by $d_{j}\left(t\right)=∥p_{u}\left(t\right)-p_{v_{j}}\left(t\right)∥$. The mobility of the UAV and vehicles creates a dynamic network where the Euclidean distance $d_{j}\left(t\right)$ varies over time. To maintain link quality, the UAV adjusts its position to reduce this distance and meet the required QoS targets.

### 3.1. Network Architecture

**Figure 3:**
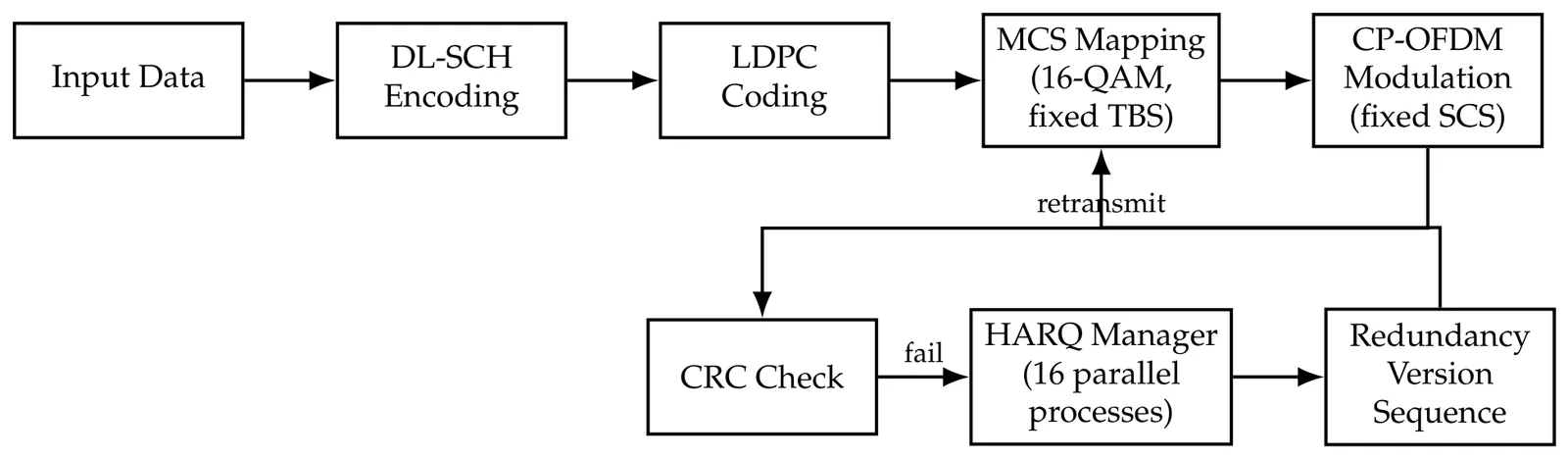
Downlink physical-layer processing chain. OFDM modulation follows 3GPP TS 38.211 [29]; DL-SCH/LDPC encoding and rate matching follow 3GPP TS 38.212 [30]; MCS/TBS selection follows 3GPP TS 38.214 [27]; the HARQ process management (16 parallel processes, CRC-triggered retransmission, fixed RV sequence) follows 3GPP TS 38.321 [31].

The considered network architecture consists of a UAV operating as a flying BS and providing downlink connectivity to multiple vehicles. The communication system models the physical downlink shared channel (PDSCH), which is the primary 5G NR channel used to deliver user-plane data and associated control information [27]. Specifically, the PDSCH carries the transport blocks generated by the downlink shared channel coding (DLSCH) after low density parity check (LDPC) encoding, modulation and coding scheme (MCS) mapping, and HARQ processing, as illustrated in Figure 3. In our system, it represents the physical-layer resource over which the UAV delivers scheduled data to each vehicle in every transmission time interval. To evaluate the impact of UAV trajectory adaptation on communication performance, the UAV incorporates a standard-compliant 5G communication stack based on 3GPP specifications [28], as illustrated in Figure 2b.

The physical layer addresses downlink transmission via conventional cyclic-prefix orthogonal frequency-division multiplexing (OFDM) with a fixed subcarrier spacing (SCS), following the waveform and numerology specified in 3GPP TS 38.211 [29]. Figure 3 summarizes the resulting processing chain. Input data are encoded into transport blocks via the DLSCH using LDPC coding, including transport-block CRC attachment, code-block segmentation, and rate matching, as defined in 3GPP TS 38.212 [30]. To isolate the gains provided by the trajectory optimization from the effects of dynamic link adaptation, a fixed MCS is employed, specifically 16-QAM with a constant transport block size (TBS), in accordance with the MCS and TBS determination procedures of 3GPP TS 38.214 [27]. Reliability is maintained through a HARQ mechanism with 16 parallel processes, where retransmissions are triggered by cyclic redundancy check (CRC) failures using a fixed redundancy version sequence, consistent with the HARQ entity management defined in 3GPP TS 38.321 [31].

The data-link layer comprises the packet data convergence protocol (PDCP), radio link control (RLC), and MAC sublayers. The PDCP performs header compression and packet processing, while the RLC handles segmentation and reassembly. Resource allocation is modeled at the link level: at each optimization interval, the communication performance of each UAV–vehicle link is evaluated independently, with the full PDSCH resource set allocated to that link throughout the evaluation. Consequently, multiuser scheduling, inter-user resource sharing, and the resulting intra-cell interference are not modeled. This controlled abstraction isolates the impact of UAV positioning on individual radio-link quality, allowing variations in throughput and reliability to be attributed to changes in UAV positioning and channel conditions rather than dynamic resource allocation or link adaptation. The model therefore does not aim to reproduce the complete functionality of a practical 5G NR deployment, where multiple users are multiplexed within each frame and adaptive modulation and coding are employed. Instead, it provides a controlled per-link baseline for isolating and quantifying the communication gains achieved through UAV trajectory optimization.

At the network layer, radio resource control (RRC) procedures are incorporated to represent essential radio resource management and signaling functions. Finally, the application layer executes the proposed UAV trajectory adaptation algorithm. This layer receives real-time vehicle position updates through the TraCI interface and determines the UAV motion commands subject to velocity and acceleration constraints. The resulting architecture enables a tight coupling between microscopic vehicular mobility and link-level communication performance, facilitating the evaluation of UAV-assisted vehicular networks under realistic operating conditions.

### 3.2. Propagation Channel

The wireless communication channel between the UAV and each vehicle is modeled using the CDL fast-fading model, a geometry-based stochastic channel model standardized by 3GPP for 5G NR performance evaluation [32,33]. The CDL model represents the propagation environment through discrete multipath clusters characterized by delays, average powers, and angular profiles, thereby capturing both large-scale effects (path loss and log-normal shadowing) and small-scale fading caused by multipath propagation and user mobility. By adopting the standardized CDL framework, the simulated radio environment closely follows 3GPP specifications validated through measurement campaigns. Nevertheless, directional beamforming, antenna array geometry, and beam management procedures are beyond the scope of this work. Instead, antenna radiation patterns, polarization, and spatial characteristics are inherently incorporated into the CDL channel formulation.

The highway scenario considered in this work is modeled as a RMa environment following 3GPP TR 38.901 [32]. Since the serving node is an aerial UAV rather than a conventional terrestrial base station, the standard RMa model is extended according to the UAV channel specifications of 3GPP TR 36.777 [33]. These extensions incorporate altitude-dependent propagation characteristics while preserving the standardized RMa framework for frequencies at 0.5 GHz to 100 GHz. Accordingly, the large-scale propagation model considers both LOS and non-line of sight (NLOS) conditions using the standardized aerial path loss formulations described below.

The large-scale propagation model follows the 3GPP RMa channel specification for UAV communications [32,33]. In particular, the propagation conditions account for both line-of-sight (LOS) and non-line-of-sight (NLOS) links, where the corresponding occurrence probabilities and terrestrial-to-aerial transition models are defined according to the 3GPP recommendations. The LOS path loss is expressed as

(1) $$PL_{\mathrm{LOS}}^{\mathrm{aer}}=\max\left(23.9-1.8\log_{10}\left(z_{u}\right),20\right)\log_{10}\left(d_{j}\right)+20\log_{10}\left(\frac{40\pi f_{c}}{3}\right),$$

where $PL_{\mathrm{LOS}}^{\mathrm{aer}}$ denotes the aerial LOS path loss model, as specified in [32,33]. Parameter $f_{c}$ is the carrier frequency in GHz,$d_{j}$ is the distance between the UAV and the *j*-th vehicle, and $z_{u}$ is the altitude of the UAV. For NLOS propagation, the aerial path loss is given by

(2) $$PL_{\mathrm{NLOS}}^{\mathrm{aer}}=\max\left(PL_{\mathrm{LOS}}^{\mathrm{aer}},-12+\left(35-5.3\log_{10}\left(z_{u}\right)\right)\log_{10}\left(d_{j}\right)+20\log_{10}\left(\frac{40\pi f_{c}}{3}\right)\right).$$

Shadowing is modeled through an additive Gaussian term $N(0,\sigma_{\mathrm{SF}}^{2})$ in the path loss expression. In UAV-assisted networks, the shadowing standard deviation $\sigma_{\mathrm{SF}}$ varies with UAV altitude and differs for LOS and NLOS conditions [33]. Small-scale fading is modeled according to the 3GPP CDL procedure for UAV-assisted RMa environments. The model generates multipath clusters with their corresponding delays, powers, angular characteristics, polarization properties, and channel coefficients, after which the large-scale attenuation and shadowing are incorporated to obtain the complete channel realization.

### 3.3. Communication Metrics

The evaluated 5G NR communication metrics include the SNR, throughput Th (bps), and BLER. Following the resource element (RE)-based SNR definition adopted in the MATLAB 5G Toolbox link-level simulations, the SNR is computed as

(3) $$\mathrm{SNR}=\frac{S_{\mathrm{RE}}}{N_{\mathrm{RE}}},$$

where $S_{\mathrm{RE}}$ and $N_{\mathrm{RE}}$ denote the average signal and noise powers per RE, respectively. The received signal power is normalized according to the allocation of active REs within the OFDM resource grid. For a given numerology (subcarrier spacing) and carrier bandwidth, the resource grid spans $N_{\mathrm{grid}}^{\mathrm{size}}$ resource blocks (RBs), as defined by the transmission bandwidth configuration in 3GPP TS 38.211 [29]. Since each RB consists of 12 consecutive subcarriers in the frequency domain, the total number of active subcarriers across the resource grid is given by $12N_{\mathrm{grid}}^{\mathrm{size}}$ [34]. Assuming uniform power allocation across the active subcarriers, the average received signal power per RE is

(4) $$S_{\mathrm{RE}}=\frac{P_{\mathrm{Tx}}}{PL}\frac{1}{12N_{\mathrm{grid}}^{\mathrm{size}}},$$

where $P_{\mathrm{Tx}}$ is the transmit power, PL denotes the large-scale propagation path loss, and $N_{\mathrm{grid}}^{\mathrm{size}}$ is the OFDM grid size expressed in RBs.

The receiver noise is modeled as additive white Gaussian noise (AWGN). Following the methodology adopted in the MATLAB 5G Toolbox, the noise amplitude is computed as

(5) $$N_{\mathrm{amp}}=\sqrt{\frac{kBT_{e}}{2}},$$

where *k* is the Boltzmann constant, *B* is the waveform sample rate, and $T_{e}$ is the equivalent receiver noise temperature given by

(6) $$T_{e}=T_{\mathrm{Ant}}+290\left(\mathrm{NF}-1\right),$$

where $T_{\mathrm{Ant}}$ is the antenna noise temperature and NF is the receiver noise figure expressed in linear scale.

The noise amplitude $N_{\mathrm{amp}}$ in (5) defines the standard deviation of each of the two independent (in-phase and quadrature) Gaussian components used to generate the complex AWGN samples added to the time-domain waveform. Consequently, the total average noise power is the sum of the variances of both components, $2N_{\mathrm{amp}}^{2}$. Since this noise is added in the time domain, it occupies the entire occupied bandwidth of the OFDM signal after the Fast Fourier Transform (FFT), including the FFT bins corresponding to unused subcarriers and guard bands, rather than only the subcarriers carrying data. The average noise power per RE is therefore obtained by distributing the total noise power $2N_{\mathrm{amp}}^{2}$ uniformly across all $N_{\mathrm{FFT}}$ bins of the OFDM demodulator, yielding

(7) $$N_{\mathrm{RE}}=\frac{2N_{\mathrm{amp}}^{2}}{N_{\mathrm{FFT}}},$$

where $N_{\mathrm{FFT}}$ denotes the FFT size used by the OFDM modulation. Substituting (5) into (7) yields

(8) $$N_{\mathrm{RE}}=\frac{kBT_{e}}{N_{\mathrm{FFT}}}.$$

The distinction between the total OFDM signal power and the SNR per RE arises from the different normalizations applied to the signal and noise components. While the signal power is distributed only across the active resource elements, the noise power is distributed across all FFT bins. Consequently, the effective SNR per RE depends on the resource-grid occupancy ratio $12N_{\mathrm{grid}}^{\mathrm{size}}/N_{\mathrm{FFT}}$. Furthermore, small-scale fading and antenna effects are modeled using the CDL channel model, which represents the wireless channel through multipath clusters characterized by delays, powers, and angular profiles. Parameterized by the selected delay profile (CDL-D), root-mean-square delay spread, and the relative UAV-vehicle velocity, the model also accounts for antenna polarization, radiation patterns, spatial correlation, and receive-array combining. At each simulation step, the generated time-varying channel impulse response is convolved with the transmitted OFDM waveform to obtain the received signal, from which the instantaneous received power, SNR, BLER, and throughput are computed according to (3)–(8).

At the receiver, the signal is first recovered through channel equalization, which mitigates the impairments introduced by the wireless channel (e.g., multipath fading, as modeled by the CDL model) and suppresses residual noise. The equalized soft symbols are then passed to the channel decoder, which uses the Normalized Min-Sum algorithm [35] to recover the transmitted bits. This algorithm iteratively updates log-likelihood ratios across the LDPC parity-check equations until the decoded bits satisfy the parity checks associated with the transmitted TBS, as specified in the 3GPP standard [27], or until a maximum number of iterations is reached. A transport block is declared successfully decoded if the CRC check (introduced in Section 3) passes after decoding; otherwise, it is discarded and, under the HARQ mechanism, scheduled for retransmission.

Based on this decoding outcome, we define an indicator variable $\rho_{k}\in \left\{0,1\right\}$ for each transmitted transport block *k*, where $\rho_{k}=1$ if the *k*-th transport block is successfully decoded and $\rho_{k}=0$ otherwise. This indicator is used to compute two key performance metrics: throughput and BLER. The throughput, expressed in bits per second (bps), is computed as the total amount of successfully decoded data divided by the total simulation duration:

(9) $$\mathrm{Th}=\frac{\sum_{k=1}^{N_{S}}\rho_{k}{\mathrm{TBS}}_{k}}{N_{\mathrm{Fr}}T_{\mathrm{Fr}}},$$

where ${\mathrm{TBS}}_{k}$ is the transport block size of the *k*-th block, $N_{S}$ is the total number of slots simulated, $N_{\mathrm{Fr}}$ is the total number of 5G NR frames within the simulation period, and $T_{\mathrm{Fr}}=10\;\mathrm{ms}$ is the fixed frame duration defined by the standard. The total number of slots $N_{S}$ is obtained as $N_{S}=N_{\mathrm{Fr}}S_{\mathrm{Fr}}$, where $S_{\mathrm{Fr}}$ is the number of slots per frame. Following the 3GPP NR numerology [29], the number of slots per frame depends on the subcarrier spacing through $S_{\mathrm{Fr}}=10\times 2^{\mu }$, where μ is the numerology index. In our simulations, we adopt μ=3, corresponding to a fixed SCS of 120 kHz, which yields $S_{\mathrm{Fr}}=80$ slots per frame. Since each link is evaluated independently with the full PDSCH resource allocation throughout the simulation interval, (9) represents the throughput of an individual UAV–vehicle radio link. It is bounded by the peak link rate $S_{\mathrm{Fr}}\mathrm{TBS}/T_{\mathrm{Fr}}=\mathrm{TBS}/T_{s}=75.84\;\mathrm{Mbps}$, achieved when every transport block is successfully decoded.

The second metric, BLER, quantifies the reliability of the transmission by measuring the fraction of transport blocks that are not successfully decoded out of the total number of transport blocks transmitted:

(10) $$\mathrm{BLER}=\frac{\sum_{k=1}^{N_{S}}\left(1-\rho_{k}\right)}{N_{S}}.$$

Both Th and BLER are first computed on a per-vehicle, per-slot basis, directly from the decoding outcome $\rho_{k}$ of each transport block, as defined in (9) and (10). To characterize long-term system behavior rather than instantaneous fluctuations, the results reported in Section 5 correspond to the time-averaged throughput and BLER of each vehicle over the entire simulation horizon *T*, reflecting sustained link reliability and data delivery efficiency rather than the outcome of any single slot.

These two metrics are closely tied to the SNR experienced by each vehicle. A higher SNR improves the reliability of the equalization and decoding stages, which increases the likelihood that a transport block satisfies its CRC check ($\rho_{k}=1$), thereby reducing BLER and allowing more transport blocks to contribute to the throughput sum in (9). Conversely, a lower SNR increases the probability of decoding failures, raising BLER and reducing the effective throughput even when the nominal MCS and TBS remain fixed. This SNR-throughput-BLER relationship is central to evaluating UAV-assisted vehicular networks, since it directly links the UAV’s trajectory-dependent channel conditions to the end-to-end communication performance experienced by the served vehicles.

## 4. UAV Trajectory Planning

This section introduces an optimization problem designed to guide the UAV in route planning, aiming to maximize the average SNR for each time slot. The devised solution is provided through the pseudocode of Algorithm 1.

**Algorithm 1:** UAV path planning.


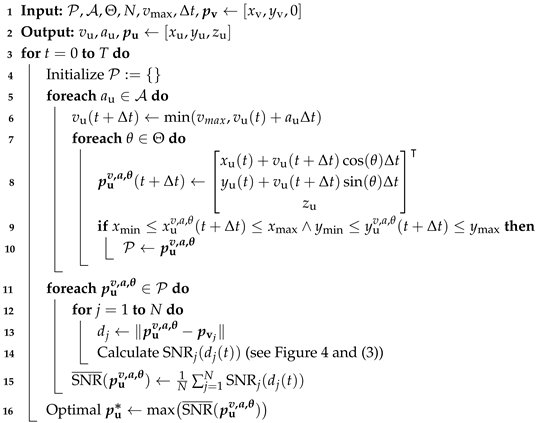




**Figure 4:**
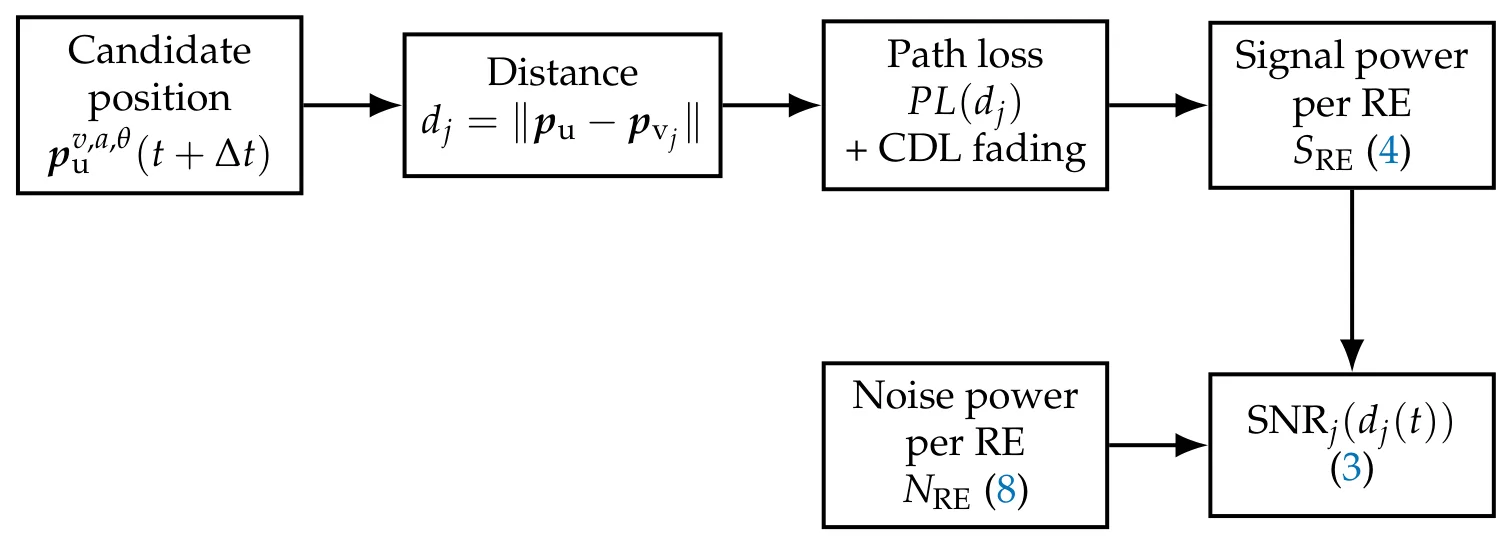
Block diagram of the per-vehicle SNR computation as a function of UAV-vehicle distance $d_{j}$, as used within Algorithm 1 (candidate position evaluation) and Equations (3)–(8).

### 4.1. UAV Decision-Making

The UAV trajectory is optimized to maximize the average SNR, ensuring continuous coverage and reliable vehicle localization at each time slot. This low-complexity framework provides robust, network-wide performance. The optimization problem is formulated as follows:

(11) $$\begin{array}{cc} \underset{p_{u},\;v_{u},\;a_{u}}{\mathrm{arg max}} & \bar{\mathrm{SNR}}(t)=\frac{1}{N}\sum_{j=1}^{N}{\mathrm{SNR}}_{j}\left(d_{j}\left(t\right)\right),\forall t\in \left[0,T\right]\\ s.t. & v_{u}\left(t+\Delta t\right)=\min(v_{\max},v_{u}\left(t\right)+a_{u}\left(t\right)\Delta t),\\ & x_{\min}\leq x_{u}\left(t\right)\leq x_{\max},\\ & y_{\min}\leq y_{u}\left(t\right)\leq y_{\max},\\ & {-a}_{\max}\leq a_{u}\left(t\right)\leq a_{\max}. \end{array}$$

The optimization problem in (11) belongs to the class of constrained nonlinear optimization problems. More generally, optimization problems involving nonlinear objective functions together with continuous and/or discrete decision variables are commonly studied within the framework of nonlinear and mixed-integer nonlinear programming (MINLP), for which comprehensive modeling and solution methodologies are available [36]. In the proposed framework, the optimization problem can be addressed using dynamic programming techniques in a recursive manner. The objective function, defined as the average SNR and denoted by $\bar{\mathrm{SNR}}$, is inherently nonlinear due to its inverse dependence on the distance $d_{j}$, a typical characteristic of wireless channel models. Additionally, the velocity constraint introduces further nonlinearity, as the min(·) function results in a piecewise formulation. The problem is inherently dynamic, as the UAV’s position $p_{u}$, velocity $v_{u}$, and acceleration $a_{u}$ evolve over the time horizon t∈[0,T]. Thus, the objective is to determine an optimal time-varying trajectory, or sequence of control actions, rather than a fixed set of values, while respecting physical and operational constraints.

Since solving the nonlinear optimization problem over the complete time horizon is computationally demanding for onboard UAV hardware, we propose a lightweight receding-horizon solution specifically designed for real-time operation under the computational constraints of UAV platforms. The search space is discretized by restricting the feasible UAV positions to a finite set of candidate locations, substantially reducing the computational burden while maintaining satisfactory trajectory performance. Given a discrete-time design, let $p_{u}$ be the UAV position to be reached based on current information in time *t* to maximize the average SNR, where $v_{u}$ represents the UAV velocity in m/s and $a_{u}$ denotes the linear acceleration in $m/s^{2}$. To handle a suboptimal but less time-consuming solution, the search space in the optimization problem is reduced by considering a finite set of possible positions P based on an angular resolution θ and velocity $v_{u}$, such that

(12) $$P=\left\{[x_{u}+v_{u}\cos\left(\theta \right)\Delta t,y_{u}+v_{u}\sin\left(\theta \right)\Delta t,z_{u}]:\theta \in \Theta \right\},$$

where the set of angular displacements from the x-axis is $\Theta =\left\{0,\Delta \theta ,2\Delta \theta ,\ldots ,2\pi -\Delta \theta \right\}$, and the velocity selected in a given discrete time interval, computed as $v_{u}\left(t+\Delta t\right)=\min\left(v_{\max},v_{u_{t}}+a_{u}\Delta t\right)$, is dependent on the maximum allowed velocity $v_{\max}$ and the set of accelerations A, defined as

(13) $$A=\left\{-a_{\max},-a_{\max}+\Delta a_{u},\ldots ,a_{\max}-\Delta a_{u},a_{\max}\right\},$$

where $a_{\max}$ denotes the UAV’s maximum allowable acceleration, obtained from the DJI drone datasheet [37]. Next, we outline the key implementation aspects required for deploying the proposed algorithm.

### 4.2. Algorithm Analysis

The proposed algorithm computes the optimal UAV trajectory by maximizing the average SNR at each time step, assuming that the UAV has access to the instantaneous positions of all active vehicles. In the simulation framework, this information is obtained through the SUMO-TraCI interface, which provides synchronized mobility updates. After reading the input variables, the application runs for the full simulation horizon *T*. It first initializes the set of candidate positions P (line 4), then generates tentative UAV positions $p_{u}^{v,a,\theta }$ by enumerating combinations of accelerations and angular displacements (lines 5–10). Each combination yields a future velocity and the corresponding predicted position, which is added to P. Next, all positions in P are evaluated (lines 11–15). For each candidate, the distance $d_{j}$ to every vehicle is computed, and the corresponding ${\mathrm{SNR}}_{j}\left(d_{j}\right)$ values are obtained following the computation chain illustrated in Figure 4, which maps the UAV-vehicle distance to the path loss and CDL small-scale fading, the resulting signal and noise power per RE, and finally the per-vehicle SNR according to (3)–(8). The average SNR across covered vehicles is then calculated. Finally, the algorithm selects the position $p_{u}^{*}$ that maximizes the average SNR (line 16). The full procedure is summarized in Algorithm 1.

The computational complexity of Algorithm 1 is given by O(T·A·Θ·N). At each time step, the algorithm evaluates all candidate UAV positions generated by the discretized acceleration A and angular Θ sets, computes the corresponding distances and SNRs for all *N* users, and selects the candidate that maximizes the average SNR. This polynomial scaling ensures that the motion-planning procedure remains computationally tractable, thereby enabling low-latency execution and real-time decision-making. Although the optimization follows a greedy strategy, the search space is restricted to physically reachable states defined by the UAV kinematic constraints, ensuring smooth trajectories that remain consistent with the dynamics of practical aerial platforms.

The adopted design reflects a deliberate trade-off between local greedy optimization and global trajectory planning. While a global optimization strategy could provide a tighter performance bound, its practical implementation would require perfect a priori knowledge of future vehicular trajectories, which represents an assumption that is unrealistic in highly dynamic highway environments. By contrast, the proposed local search strategy allows the UAV to adaptively respond to microscopic mobility variations and instantaneous communication requirements. Moreover, the framework jointly accounts for communication objectives and physical feasibility by enforcing strict kinematic constraints, including maximum acceleration and velocity limits. This ensures that the computed trajectory remains compatible with the computational and operational constraints of practical UAV platforms.

## 5. Performance Evaluation

This section describes the simulation setup, which integrates a 5G communication model using the 3GPP CDL-D channel and evaluates performance through key QoS metrics such as throughput and BLER. Two traffic conditions are analyzed: a single-vehicle case resembling a tracking scenario and a multi-vehicle setting capturing diverse mobility patterns. A hovering UAV, one following a predefined trajectory, and a centroid-based UAV are included to benchmark and validate the proposed method. All reported results are averaged over 20 independent simulation realizations with different random seeds for each of the five evaluated scenarios, accounting for the stochastic nature of vehicle mobility and wireless channel conditions.

### 5.1. Simulation Setup

**Figure 5:**
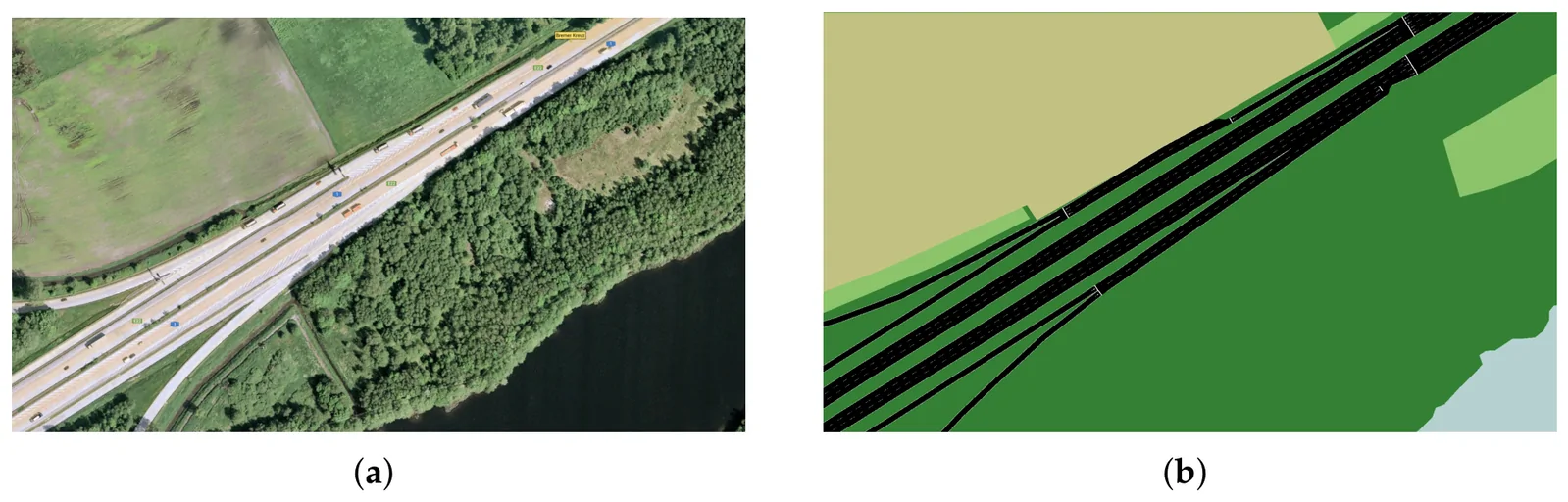
Highway test scenario in Bremen, Germany [9]. (**a**) Satellite view. (**b**) Map uploaded to SUMO.

The simulation models a highway scenario where UAVs provide 5G connectivity to vehicles. Mobility is generated in SUMO, while UAV positioning and communication are handled in MATLAB using the 5G Toolbox. Both platforms exchange information via TraCI, with MATLAB as the client and SUMO as the server. The setup consists of an 800m two-way highway segment in Bremen with four lanes per direction, as shown in Figure 5. The lower section carries left-to-right traffic with three entrances and one exit; the upper section carries right-to-left traffic with one entrance and three exits. UAV-vehicle communication follows the architecture in Section 3.1, and additional parameters appear in Table 2.

The main simulation parameters listed in Table 2 were selected to represent a realistic UAV-assisted vehicular communication scenario. The carrier frequency (26 GHz), 50 MHz bandwidth, and numerology μ=3 (120 kHz SCS) follow the 3GPP NR FR2 specifications and are consistent with the CDL-D channel model adopted in this work [29,32,33]. The 800m highway enables representative vehicle mobility while remaining within the effective coverage region of a single UAV. The maximum vehicle and UAV speeds (20 m/s and 23m/s, respectively) reflect realistic highway operation, allowing the UAV to effectively track vehicle mobility. Finally, the UAV altitude of 100m provides predominantly LOS propagation while complying with typical low-altitude UAV operation limits [38].

Within this setup, the PDSCH and CDL-D models play complementary roles. The PDSCH governs transport block transmission, decoding, and HARQ retransmissions, thereby determining the throughput and BLER for a given SNR. The CDL-D model captures the propagation channel between the UAV and the *j*-th vehicle, including dominant LOS propagation, multipath fading, and antenna effects. As the UAV and vehicles move, the resulting time-varying channel response produces the instantaneous SNR, which directly determines the PDSCH decoding performance and the corresponding throughput and BLER reported in the following results.

At the midpoint of the scenario, all UAVs are initially placed to ensure full coverage. “UAV4: centroid” adjusts its position toward the centroid of the active vehicles in the simulation area [39], relying on vehicle position information obtained through periodic reporting or UAV-based sensing. “UAV3: static” remains fixed at its initial position, and “UAV2: predefined” follows a constant-speed (20 m/s) back-and-forth trajectory centered between the two main avenues. “UAV1: proposed” dynamically adapts its motion according to Algorithm 1 driven by average SNR maximization. Vehicles move at speeds up to 20 m/s, and their spatial distribution along the highway follows a Poisson process with density λ vehicles per kilometer per lane [40], giving $P\left(N\right)=\frac{\lambda^{N}e^{-\lambda }}{N!}$. Specifically, the simulated traffic volumes correspond on average to 23 and 83 vehicles for the one-way low- and high-density scenarios, respectively, and to 42 and 123 vehicles for the two-way low- and high-density scenarios, respectively.

### 5.2. Highway Scenario: Single Vehicle

This scenario, which aims to replicate a vehicle tracking case, involves a single vehicle starting from the main avenue in the lower section of the road, moving from left to right.

#### 5.2.1. Sensitivity Analysis

**Figure 6:**
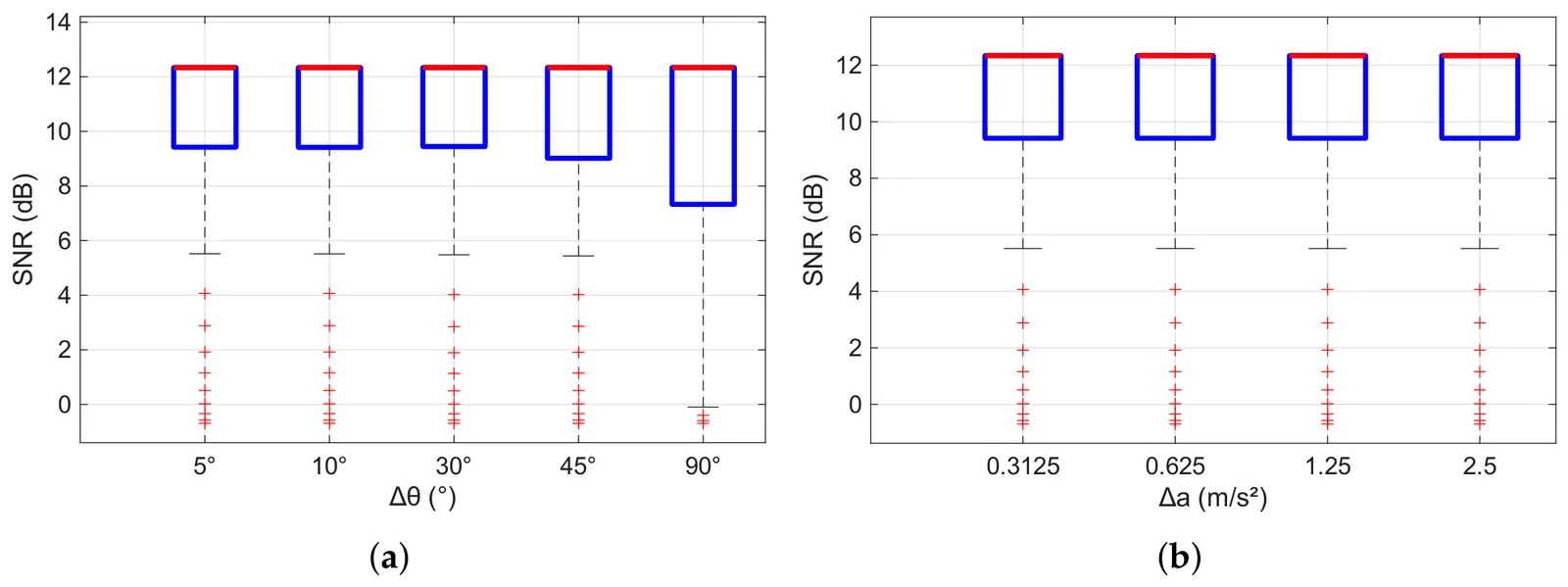
Sensitivity analysis of the proposed algorithm for (**a**) the angular displacement step Δθ and (**b**) the acceleration step $\Delta a_{u}$ for the single-vehicle scenario.

In order to evaluate the sensitivity of the proposed algorithm to the parameters that define the search space, we analyze the impact of the angular displacement step Δθ and the acceleration step $\Delta a_{u}$ on the average SNR achieved by “UAV1: proposed”. A smaller angular displacement step allows for more granular directional adjustments, enhancing the UAV’s ability to optimize its trajectory and maintain a stronger communication link with the vehicle. As shown in Figure 6a, varying Δθ within the range 5° to 90° results in a nearly unchanged median SNR of 12.34 dB. However, the dispersion of the SNR increases with coarser angular resolution. Specifically, the interquartile range remains at 2.92 dB for $\Delta \theta =5^{\circ },10^{\circ }\;\mathrm{and}\;30^{\circ }$, but expands to 5 dB when $\Delta \theta =90^{\circ }$, indicating greater variability under larger angular step sizes.

As illustrated in Figure 6b, the impact of acceleration resolution on the resulting SNR is negligible, indicating that increasing the cardinality of the acceleration set does not yield a meaningful improvement in the average SNR. Although a finer acceleration step provides more granular velocity adjustments, its benefit depends strongly on the mobility dynamics of the scenario. In the considered setup, vehicles move at a constant speed; consequently, the UAV primarily performs two phases: an initial transient phase in which it accelerates to approach the target vehicle, and a steady-state phase in which it approximately matches the vehicle’s velocity to maintain favorable link geometry and maximize SNR. Since the steady-state dominates the simulation time, additional acceleration granularity offers limited performance gains. This explains the marginal influence of acceleration resolution on the observed SNR results. These finding allow us to select $\Delta \theta =10^{\circ }$ and $\Delta a_{u}=1.25\;m/s^{2}$ for the subsequent performance evaluation, as they provide a good balance between computational complexity and SNR performance.

#### 5.2.2. Tracking Evaluation

**Figure 7:**
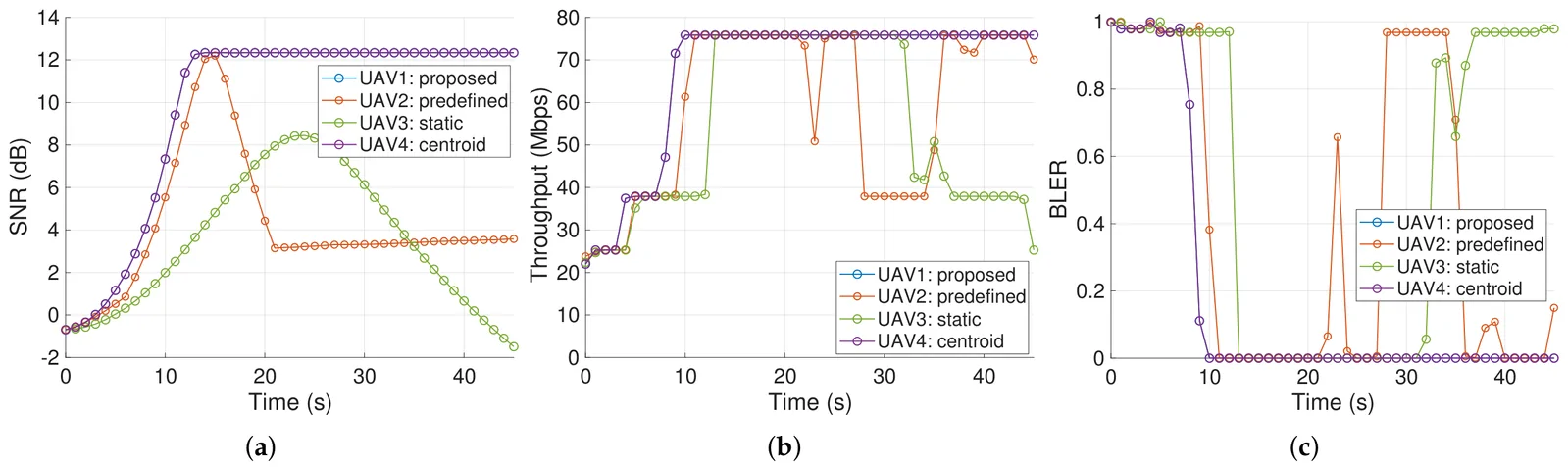
Single-vehicle highway scenario (tracking). (**a**) SNR at the receiver side. (**b**) Throughput. (**c**) BLER.

The performance of the four UAV strategies is shown in Figure 7, where “UAV1: proposed” and “UAV4: centroid” exhibit the highest SNR, peaking at 12.3 dB and at 14 s as they approach and then hover above the vehicle. “UAV2: predefined” peaks at 12 dB at 15 s, then decreases as it moves away, before realigning with the vehicle at 21 s. “UAV3: static” exhibits a bell-shaped curve, reaching 8.4 dB at 24 s as the vehicle first approaches and later departs from its fixed position.

The disparate movement behavior exhibited by the UAVs is manifest in the outcomes obtained for throughput, as shown in Figure 7b, and BLER, as presented in Figure 7c. An evident relationship is observed between SNR, throughput, and BLER, as expected: higher SNR values result in increased throughput and a corresponding reduction in BLER. It is noteworthy that at 10 s, a maximum throughput of 75.8 Mbps is achieved, and the BLER is reduced to zero, underscoring the negative correlation between these metrics. The proposed and centroid-tracking approaches exhibit similar behavior, as both converge toward the vehicles’ spatial distribution. This is expected under the considered propagation conditions, where SNR is largely distance-driven due to the high LOS probability in the RMa scenario, making SNR maximization effectively equivalent to approaching the geometric centroid. Consequently, both methods yield comparable performance. It is important to note that both rely on vehicle position information; however, while the centroid method applies a purely geometric rule, the proposed approach optimizes a communication-driven metric, providing a more general formulation that can better adapt to more complex channel conditions or heterogeneous requirements.

### 5.3. Highway Scenario: Multiple Vehicles

The multi-vehicle scenarios emulate realistic road conditions under two traffic patterns: a single-direction flow representing peak commuting hours, and a bidirectional flow reflecting typical daily operation. Each pattern includes low- and high-density cases, defined by their respective λ values.

#### 5.3.1. One-Way Traffic: Low Vehicular Density

The lower section of the roadway is employed to simulate low-density traffic traveling in a unidirectional manner, as shown in Figure 5. This objective was attained by establishing a vehicle density parameter of λ=0.3 for the primary avenue and λ=0.15 for the two converging secondary streets. This methodology was implemented to prevent congestion at the intersection.

**Figure 8:**
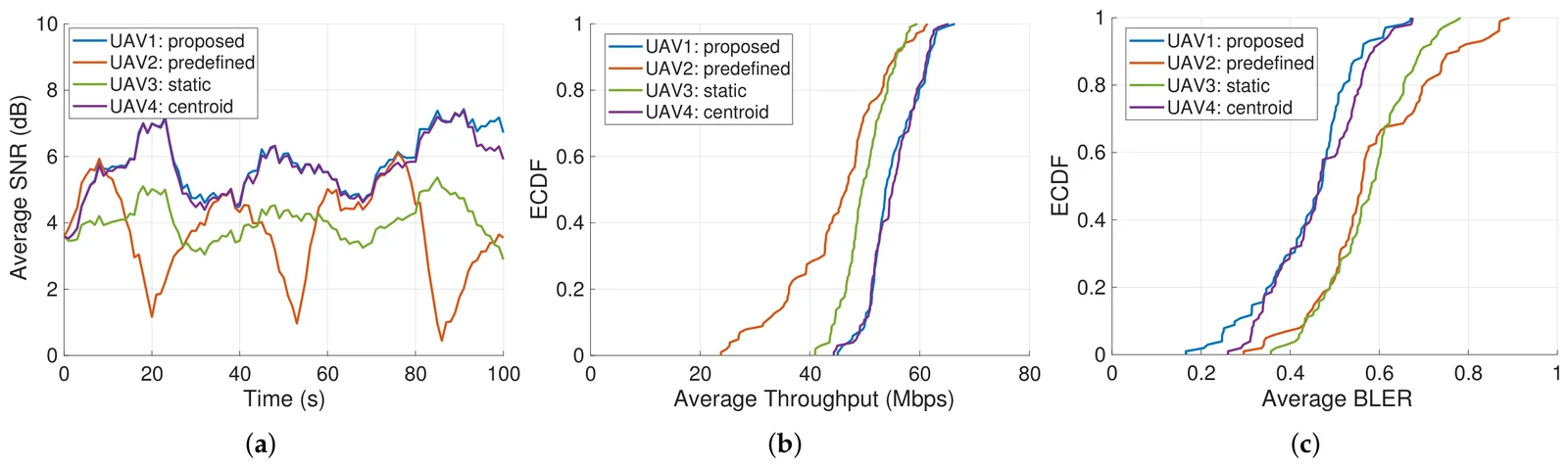
One-way low-density traffic scenario. (**a**) Average SNR. (**b**) ECDF of average throughput. (**c**) ECDF of BLER.

Performance, in terms of average SNR and the empirical cumulative distribution function (ECDF) of throughput and BLER, is shown in Figure 8. The ECDF complements the average performance metrics by revealing the distribution of link quality across vehicles and simulation runs, thereby highlighting differences in typical performance and the occurrence of low throughput or high BLER cases that may not be apparent from the mean values alone. As seen in Figure 8a, the proposed UAV1 consistently achieves the highest SNR, with a mean of 5.79 dB and a standard deviation of 0.93 dB. UAV2 attains the lowest mean SNR of 3.75 dB with greater variability (1.37 dB), while UAV3 yields a mean of 4.03 dB and a standard deviation of 0.58 dB. In this low-density scenario, larger gaps between vehicles amplify the impact of individual link qualities on UAV1’s decisions, yielding mean SNR improvements of 2.04 dB and 1.76 dB over UAV2 and UAV3, respectively. The difference between UAV1 and UAV4 is only 0.14 dB, indicating that, under these high-LOS conditions, the two strategies achieve effectively equivalent link quality.

UAV1 also delivers the highest throughput, shown by the rightward shift of its ECDF curve in Figure 8b. At the 90th percentile, UAV1 reaches 61.7 Mbps, outperforming UAV2 (55.5 Mbps), UAV3 (55.72 Mbps), and UAV4 (61.31 Mbps) by 11.2%, 10.7%, and 0.6%, respectively. Conversely, UAV1 achieves the lowest BLER, reflected by the leftward shift of its curve in Figure 8c. At the 90th percentile, UAV1 attains a BLER of 0.56, whereas UAV2, UAV3, and UAV4 reach 0.77, 0.69, and 0.59, respectively. These higher BLER levels stem from the influence of vehicles located farther from the UAVs, typically near the edges of the map.

#### 5.3.2. One-Way Traffic: High Vehicular Density

**Figure 9:**
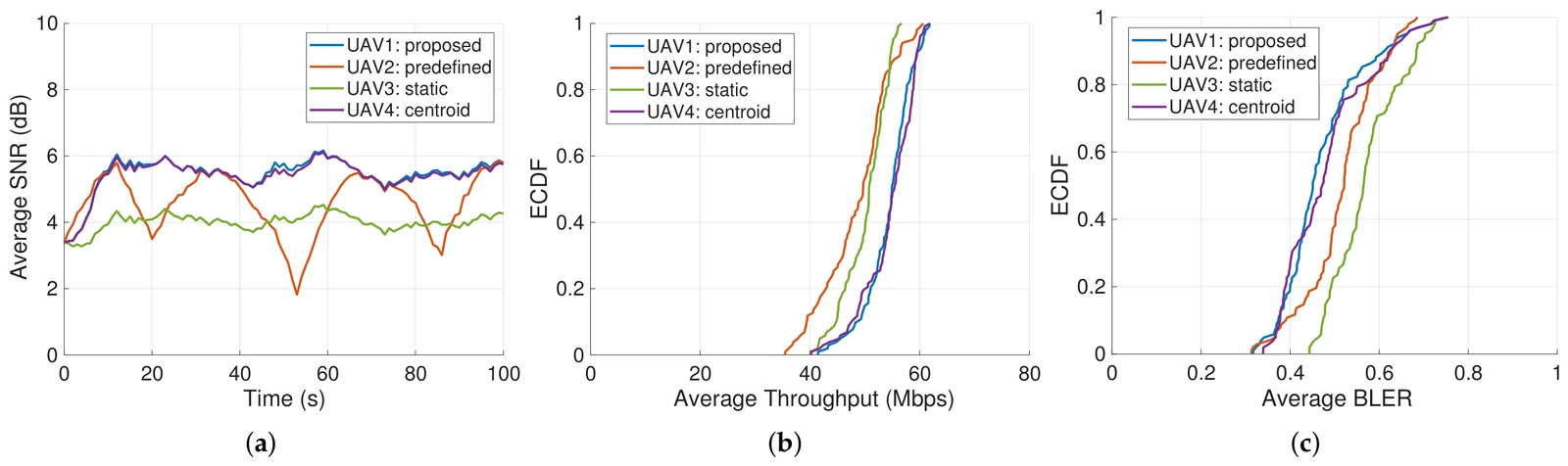
One-way high-density traffic scenario. (**a**) Average SNR. (**b**) ECDF of average throughput. (**c**) ECDF of BLER.

In this instance, a higher vehicle density has been considered, with a value of λ=1.2 applied to the main avenue and λ=0.35 to the two secondary streets in the lower section of the road. As illustrated in Figure 9a, the SNR of the proposed UAV1 is predominantly higher, with a mean of 5.46 dB and a standard deviation of 0.54 dB. In contrast, UAV2 presents a mean of 4.61 dB with a standard deviation of 0.9 dB, exhibiting more substantial fluctuations over time. The hovering UAV3 exhibits the lowest average SNR, with a mean of 4.01 dB and a standard deviation of 0.26 dB, indicating reduced variations compared to the other configurations. Finally, UAV4 achieves performance comparable to UAV1, with a mean SNR of 5.41 dB and a standard deviation of 0.52 dB. This indicates that both approaches provide similar link-quality performance, particularly under high LOS probability conditions where distance dominates the channel behavior.

A similar pattern is observed for the average throughput, with UAV1 consistently outperforming UAV2 and UAV3 across all probability levels, while exhibiting a comparable trend to that of UAV4, as shown in Figure 9b. At the 50th percentile, UAV1, UAV2, UAV3, and UAV4 achieve 54.93 Mbps, 49.68 Mbps, 50.77 Mbps, and 55.36 Mbps, respectively. This advantage remains evident at higher percentiles: at the 90th percentile, UAV1 reaches 59.35 Mbps, compared with 56.16 Mbps for UAV2 and 54.63 Mbps for UAV3, corresponding to gains of 5.7% and 8.6%, respectively. As shown in Figure 9c, the behavior of UAV1 is similar to that of UAV4, while maintaining the lowest BLER across the full probability range compared with UAV2 and UAV3. At the 90th percentile, UAV1, UAV2, UAV3, and UAV4 record BLER values of 0.61, 0.63, 0.68, and 0.62, respectively, corresponding to reductions of 3.2%, 10.3%, and 1.6% relative to UAV2, UAV3, and UAV4. These results confirm the reliability improvement achieved by the proposed UAV1 strategy.

#### 5.3.3. Two-Way Traffic: Low Vehicular Density

**Figure 10:**
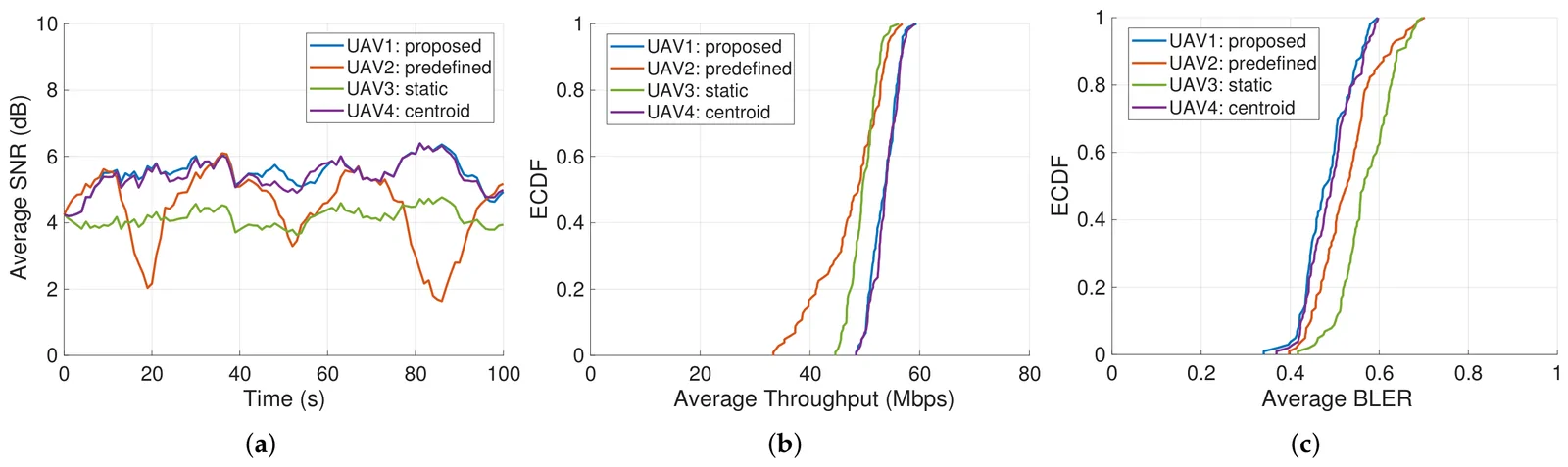
Two-way low-density traffic scenario. (**a**) Average SNR. (**b**) ECDF of average throughput. (**c**) ECDF of BLER.

This section adopts the same traffic parameters as the low-density traffic, one-way scenario in the lower area: λ=0.3 on the main avenue and λ=0.15 on the two converging secondary streets. In the upper area, comprising one entrance and three exits, each route is assigned λ=0.2. As shown in Figure 10a, UAV1 achieves the highest SNR throughout the simulation, with a mean of 5.48 dB and a standard deviation of 0.47 dB, indicating stable link quality. UAV2 follows with a mean SNR of 4.44 dB and greater variability (1.11 dB), while UAV3 records 4.15 dB with a standard deviation of 0.28 dB. In comparison, UAV4 achieves a mean SNR of 5.42 dB with a standard deviation of 0.47 dB, exhibiting performance close to the proposed approach. Overall, UAV1 provides SNR gains of 1.04 dB over UAV2 and 1.33 dB over UAV3, while maintaining performance comparable to the centroid-based UAV4 strategy. These results indicate that the proposed method achieves near-optimal link quality, with performance similar to centroid tracking under the considered conditions.

The behavior of the average throughput is illustrated through the ECDF in Figure 10b. At the 90th percentile, UAV1 achieves 56.70 Mbps, compared with 53.96 Mbps, 52.80 Mbps, and 56.57 Mbps for UAV2, UAV3, and UAV4, respectively. These results show that UAV1 outperforms UAV2 and UAV3 by 2.74 Mbps and 3.90 Mbps, respectively, while exhibiting performance very close to that of UAV4, with only a marginal difference of 0.13 Mbps. This indicates that both UAV1 and UAV4 achieve similar throughput levels, although UAV1 maintains slightly more consistent performance. A similar trend is observed for BLER, as shown in Figure 10c. At the 90th percentile, UAV1 records a BLER of 0.56, compared with 0.63, 0.64, and 0.57 for UAV2, UAV3, and UAV4, respectively. This corresponds to relative reductions of approximately 11.1% and 12.5% with respect to UAV2 and UAV3. While UAV4 exhibits reliability close to the proposed method, UAV1 maintains the lowest BLER across the analyzed percentiles, confirming the improved link reliability provided by the proposed strategy.

#### 5.3.4. Two-Way Traffic: High Vehicular Density

**Figure 11:**
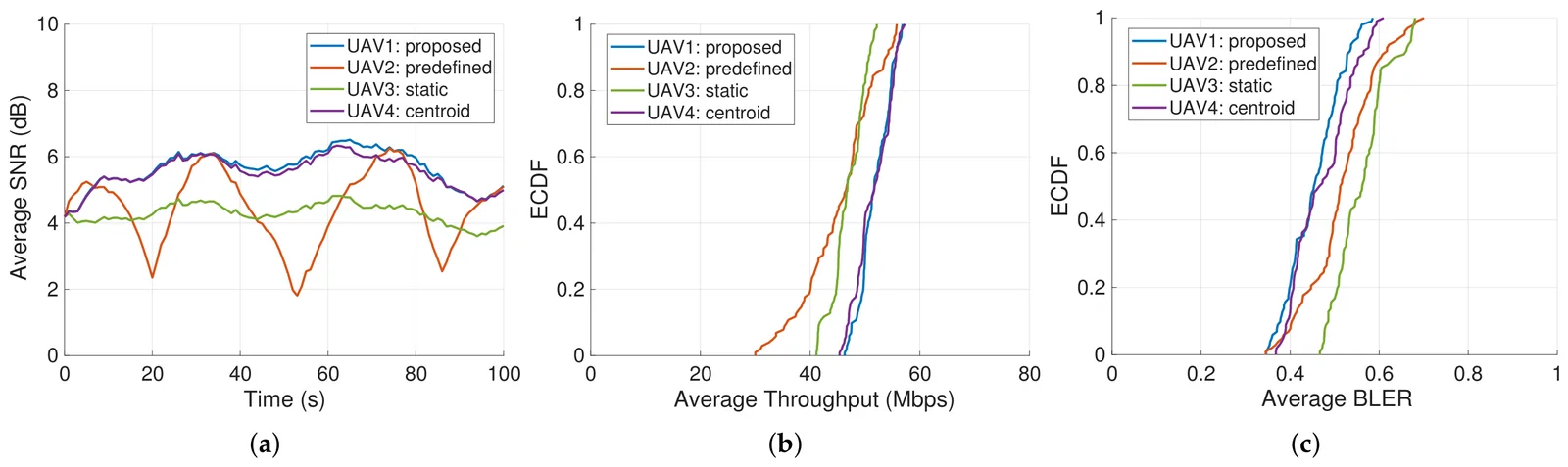
Two-way high-density traffic scenario. (**a**) Average SNR. (**b**) ECDF of average throughput. (**c**) ECDF of BLER.

In this simulation, the lower section considers a central avenue with λ=1 and secondary streets with λ=0.3. In the upper section, the main exit, corresponding to the straight-line traffic flow, has λ=0.7, while the two secondary exits have λ=0.35. Figure 11a shows that UAV1 maintains the highest average SNR values throughout the simulation, with a mean of 5.63 dB and a standard deviation of 0.56 dB. UAV4 follows closely with a mean SNR of 5.52 dB and a standard deviation of 0.50 dB, indicating performance comparable to the proposed strategy. In contrast, UAV2 records a lower mean SNR of 4.48 dB with a standard deviation of 1.12 dB, reflecting greater temporal variability. Finally, UAV3 achieves a mean SNR of 4.29 dB and a standard deviation of 0.30 dB. Overall, UAV1 provides SNR gains of 1.15 dB and 1.34 dB over UAV2 and UAV3, respectively, while maintaining a slight advantage of about 0.11 dB over UAV4, confirming the effectiveness of the proposed positioning strategy under high vehicular density.

In Figure 11b, the ECDF of the average throughput shows that UAV1’s curve is slightly shifted to the right, indicating consistently higher throughput across the distribution. At the 90th percentile, UAV1 achieves 55.56 Mbps, compared with 53.83 Mbps, 50.70 Mbps, and 55.64 Mbps for UAV2, UAV3, and UAV4, respectively. These results indicate that UAV1 provides throughput gains of 1.73 Mbps and 4.86 Mbps over UAV2 and UAV3, while maintaining performance comparable to UAV4, with only a marginal difference of 80 kbps. Overall, UAV1 and UAV4 achieve the highest throughput levels, although UAV1 maintains slightly more stable behavior across the distribution. A similar trend is observed for the BLER distribution in Figure 11c. At the 90th percentile, UAV1 reaches 0.53, improving upon UAV2 (0.62) and UAV3 (0.66), while remaining close to UAV4 (0.56). The steeper ECDF curve of UAV1 also suggests lower variability in the BLER distribution. Overall, these results confirm that the proposed UAV1 strategy provides the best reliability under high vehicular density, while UAV4 offers competitive but slightly inferior performance.

### 5.4. Statistical Analysis

Table 3 summarizes the statistical performance of the evaluated UAV deployment strategies across all scenarios. Overall, the proposed UAV1 approach consistently achieves the highest mean SNR, ranging from 5.46 dB to 5.79 dB in the one-way scenarios and from 5.48 dB to 5.63 dB in the two-way scenarios, while maintaining moderate variability. These favorable signal conditions translate into improved reliability, as UAV1 systematically attains the lowest BLER percentiles, with $P_{90}$ values between 0.53 and 0.61, outperforming the predefined (UAV2) and static (UAV3) baselines. In terms of throughput, UAV1 also delivers competitive median and high-percentile performance, with reduced dispersion compared with UAV2, as reflected by smaller interquartile ranges (IQRs), indicating a more stable user experience. Interestingly, the centroid-based strategy (UAV4) exhibits throughput and reliability metrics that closely match those achieved by UAV1 in several scenarios. This behavior can be attributed to the characteristics of the RMa propagation environment, where LOS conditions are predominant. Under such conditions, both maximizing the SNR and following the traffic centroid tend to place the UAV in locations that reduce the average distance to the vehicles. Consequently, the channel-aware optimization performed by UAV1 often converges to positions close to the geometric centroid, leading to similar performance trends between UAV1 and UAV4. Nevertheless, UAV1 maintains a slight but consistent advantage in SNR and BLER, highlighting the benefit of explicitly optimizing the communication channel conditions. Moreover, the proposed formulation is more general, as it directly optimizes a communication metric and can accommodate more complex channel conditions, user prioritization, or non-uniform propagation environments, where the centroid is no longer optimal.

### 5.5. Implementation Scope and Limitations

The proposed framework operates at a carrier frequency of 26 GHz, within the FR2 band, and adopts a defined level of physical-layer abstraction. Rather than explicitly modeling radio frequency (RF) hardware impairments, such as phase noise, power-amplifier nonlinearities, or beamforming codebook implementation, the framework employs a 3GPP-compliant link-level representation based on the RMa path loss and CDL-D fast-fading models, together with the PHY processing chain described in Section 3.1. The detailed formulations and propagation characteristics of these models are presented in the preceding sections. This abstraction is sufficient to capture the channel variations relevant to UAV trajectory adaptation and to obtain the SNR, throughput, and BLER metrics reported in Section 5, while avoiding additional RF-level complexity. It also keeps the computational cost tractable, since the trajectory optimization requires repeated evaluation of the communication link for multiple candidate UAV positions at every simulation time step. Furthermore, key performance metrics including SNR, throughput, and BLER are evaluated, providing complementary insights into link quality, achievable data rates, and reliability, respectively, while maintaining tractable simulation complexity.

To isolate the impact of UAV trajectory optimization from antenna processing gains, the proposed framework adopts a single-antenna (1×1 SISO) configuration without directional beamforming or array gain. Consequently, the reported SNR, throughput, and BLER should be interpreted as conservative estimates under a baseline FR2 link budget. In practical deployments, directional antenna arrays and beam management would provide additional array gain, increasing the achievable SNR, throughput, and coverage while preserving the relative benefits of the proposed trajectory optimization. Similarly, the fixed 16-QAM MCS and the omission of adaptive resource allocation result in BLER values (0.4–0.6) above the operating point typically targeted by NR link adaptation (approximately 0.1). Since all UAV strategies are evaluated under the same assumptions, the reported absolute BLER values are specific to the adopted setup, whereas the relative performance improvements of the proposed method remain representative and are expected to persist under link adaptation.

The current framework does not explicitly model several key mechanisms that are central to practical mmWave deployments. In particular, the following aspects are abstracted: directional beamforming and associated array gains, radiation patterns and beamwidth control, beam alignment and tracking procedures under mobility, and beam management overhead. Instead, antenna-related effects are implicitly incorporated through the CDL channel normalization and link-level modeling assumptions, without explicitly simulating beamforming vectors or directional transmission strategies. This modeling choice reflects a deliberate trade-off between physical-layer fidelity and system-level tractability. Moreover, adopting a baseline SISO configuration allows the communication gains to be attributed to UAV trajectory optimization rather than antenna array processing. By abstracting beam management procedures, the framework isolates the impact of UAV trajectory optimization on link geometry and large-scale channel conditions, which remain primary determinants of communication performance even in directional mmWave systems. Nevertheless, in practical FR2 deployments, beam misalignment, tracking latency, and signaling overhead may introduce additional performance degradation, particularly in high-mobility vehicular scenarios. Resource allocation is modeled at the link level, with each UAV–vehicle link evaluated independently under full PDSCH allocation. Consequently, multiuser scheduling, resource sharing, and intra-cell interference are not represented. The reported throughput therefore characterizes individual link performance, explaining its limited dependence on traffic density. Since all strategies are evaluated under identical conditions, comparisons of their relative performance remain valid, while multiuser scheduling is left for future work.

The baseline strategies considered in this work, including the centroid-tracking approach, are primarily geometry-driven heuristics, whereas the proposed UAV path planning explicitly maximizes the average SNR. While the baselines provide intuitive and interpretable references, they do not represent strong channel-aware or optimization-based benchmarks. In particular, the centroid method does not account for channel dynamics or short-term communication objectives. Accordingly, the comparison should be interpreted as validation against representative heuristic strategies. To further highlight the differences between the methods, we introduce more challenging propagation conditions in which the link state between the UAV and each vehicle is deterministically classified as NLOS when the UAV-vehicle segment intersects a rectangular obstructed region of the scenario spanning x∈[900,1000] m and y∈[650,850] m in the SUMO coordinate frame, representing a localized obstruction of the highway corridor, aligned with tunnel boundaries. Outside this region, the link state is drawn according to LOS probability $P_{\mathrm{LOS}}\left(d_{2D}\right)$ given by the RMa link-state model [32] (Table 7.4.2-1), evaluated at the two-dimensional UAV-vehicle separation. Path loss is computed by using (1) or (2), shadow fading is applied as a log-normal term with $\sigma_{\mathrm{SF}}=4\;\mathrm{dB}$ for LOS links and $\sigma_{\mathrm{SF}}=8\;\mathrm{dB}$ for NLOS links, and the small-scale fading profile is selected per link according to its state, using CDL-D for LOS and CDL-A for NLOS. For the heterogeneous-QoS case, each vehicle is assigned a deterministic priority weight $w_{j}\in \left\{1,3\right\}$, with approximately 30% of vehicles designated as priority users, representing services such as public transport or emergency vehicles.

**Figure 12:**
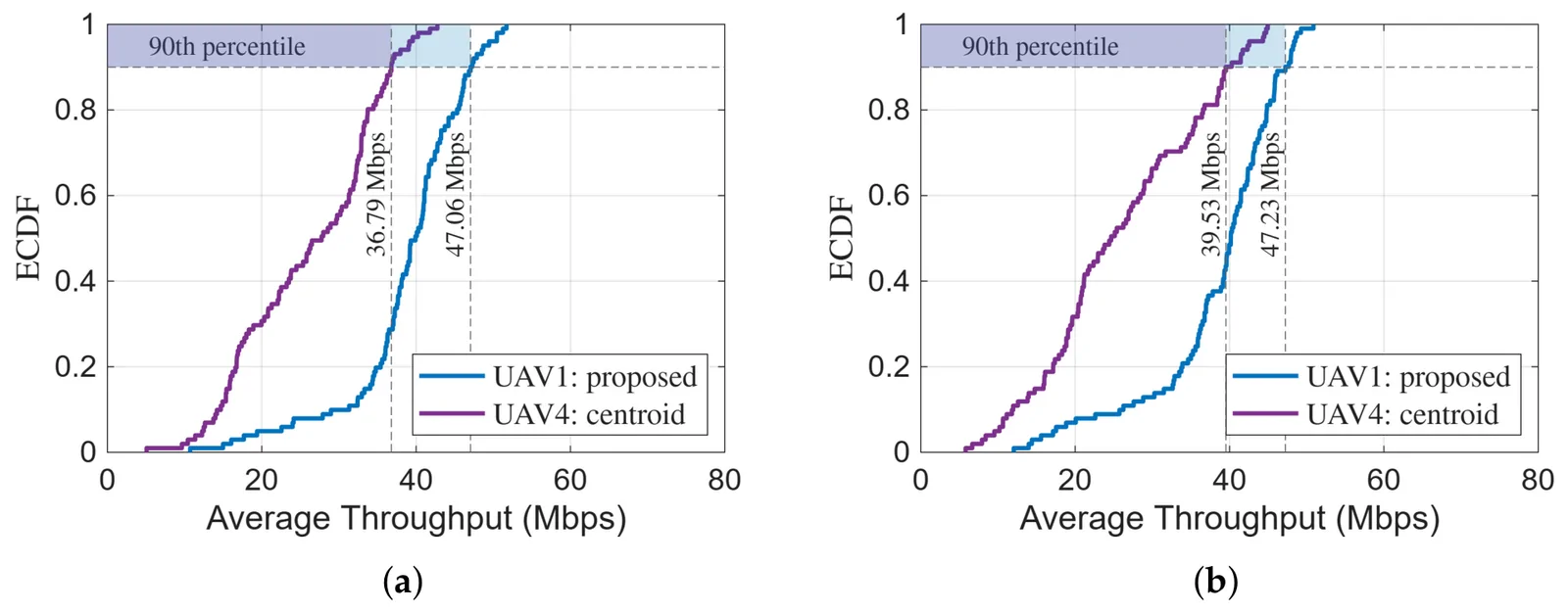
Empirical CDF of average throughput under probabilistic NLOS conditions, with the 90th percentile of each distribution indicated. (**a**) Uniform priority weights. (**b**) Priority-weighted objective.

In this setting, the objective of the proposed planner admits a weighted generalization of (11) that accommodates heterogeneous user requirements:

(14) $$\underset{p_{u},\;v_{u},\;a_{u}}{\mathrm{arg max}}\;\bar{{\mathrm{SNR}}_{w}}(t)=\frac{\sum_{j=1}^{N}w_{j}{\mathrm{SNR}}_{j}\left(d_{j}\left(t\right)\right)}{\sum_{j=1}^{N}w_{j}}$$

subject to the same kinematic constraints as in (11), where $w_{j}>0$ denotes the priority weight of the *j*-th vehicle. Setting $w_{j}=1\;\forall j$ recovers (11) exactly. The corresponding results are presented in Figure 12, where panel (a) corresponds to the uniform-weight case, maximizing the average SNR, and panel (b) to the weighted case, maximizing the priority-weighted SNR objective of (14).

From a practical deployment standpoint, the proposed trajectory planner is amenable to implementation on embedded computing platforms widely adopted in UAV systems, such as NVIDIA Jetson devices [41,42], which offer GPU-accelerated parallel processing under strict power constraints. The algorithmic structure, mainly composed of independent evaluations of UAV candidate positions, is inherently parallelizable and well suited for vectorized execution, enabling efficient utilization of onboard GPU resources. The linear complexity with respect to the number of vehicles, candidate actions, and angular sets—combined with the moderate problem scales typical of highway scenarios—suggests that the per-decision-step runtime could be tuned to meet real-time requirements. Practical deployment must account for hardware-specific factors including memory bandwidth limitations, energy consumption, and task scheduling alongside other onboard processes (e.g., perception and flight control). These aspects may introduce latency and resource contention, potentially affecting end-to-end system performance and necessitating careful system-level optimization.

## 6. Conclusions

This work presented a route-planning application integrated into the UAV’s top layer for decision-making in highway environments. Specifically, the framework operates as a discrete-motion greedy trajectory planner that dynamically adapts the UAV’s position by integrating microscopic traffic mobility (SUMO) with standard-compliant 5G communication models (MATLAB). By restricting the search space to physically reachable states based on strict kinematic constraints, such as maximum velocity and acceleration, the algorithm ensures low-latency, real-time decision-making aimed at maximizing the average received SNR. The proposed low-complexity greedy trajectory planner, applied to a 5G aerial BS, delivers consistent gains across all evaluated traffic conditions. Relative to the static UAV3 baseline, UAV1 improves the 90th-percentile throughput by up to 10.7% and reduces the 90th-percentile BLER by up to 19.7%, depending on traffic direction and density. Relative to the predefined-trajectory UAV2 baseline, these gains reach up to 11.2% in throughput and up to 27.3% in BLER at the 90th percentile. Across all scenarios, UAV1 improves the mean SNR by up to 2.04 dB over UAV2 and by up to 1.76 dB over UAV3, both in the one-way low-density scenario. These results underscore the benefits of adaptive UAV placement for enhancing QoS in dynamic vehicular networks. Relative to the centroid-tracking UAV4 baseline, the mean SNR difference does not exceed 0.14 dB, consistent with the predominance of LOS links. However, this gap widens substantially under NLOS-dominant and heterogeneous traffic conditions. Future work will explore more complex road geometries and advanced motion planning for single and cooperative UAVs, including energy-aware strategies balancing communication gains with battery life and operational endurance. In addition, we will incorporate directional antenna arrays and 3GPP-compliant beam management to evaluate the combined effects of beamforming and UAV trajectory optimization under practical FR2 deployments.

## Figures and Tables

**Table 1:** Taxonomy of related approaches in UAV-assisted vehicular networks.

Category	Ref.	Primary Objective	Key Characteristics/Limitations
Coverage Extension & Relaying	[10]	Single-/multi-UAV relaying	Mitigates RSU malfunctions to ensure high-throughput IoV links.
	[11]	Two-way mmWave relaying	Joint optimization of relay selection and transmission scheduling.
	[12,13]	Trajectory & relay selection	Multi-objective optimization (energy/delay) via continuous-space methods (e.g., Newton’s method).
	[14]	Uplink power allocation	Adjusts power in NOMA platooning to maintain SINR as vehicles diverge.
Routing, Security & Resource Management	[15]	Trust analysis & packet delivery	Utilizes artificial gorilla troop optimization for ground-to-UAV security.
	[16]	Flooding-based routing	Vehicles and UAVs cooperate to recover from path failures in urban environments.
	[17]	Energy minimization	Resource management in integrated platooning networks.
	[18]	Cooperative protocol	Allocates channel resources based on LOS probability to enhance throughput.
Edge Computing & Real-Time Data	[19]	Task sequencing	Offloads IoV application data to minimize response time in UAV-enabled MEC.
	[20]	Age of Information (AoI)	Trajectory optimization to maintain AoI for time-sensitive sensor streams.
Traffic Management & Highway Scenarios	[21]	Ad hoc routing	Shares real-time congestion data using UAVs as aerial sensors.
	[22]	Probabilistic trajectory planning	Focuses on estimating traffic density on highways.
	[23,24]	Automated inspection	Explores automated inspection and distributed WSN-assisted surveillance.
	[25,26]	3D deployment & clustering	Energy-aware deployment in complex interchanges and clustering optimization.
Proposed Framework	Ours	SNR Maximization for QoS	Integrates microscopic mobility (SUMO), discrete kinematic constraints, and 5G mmWave channels (3GPP RMa/CDL) for real-time trajectory adaptation.

**Table 2:** Simulation parameters.

Parameter	Value	Parameter	Value
Physical channel	PDSCH	Path loss scenario	RMa
Fast-fading model	CDL-D	Modulation scheme	OFDM (16-QAM)
Carrier frequency ($f_{c}$)	26 GHz	Frames ($N_{\mathrm{Fr}}$)	20–50
Total slots ($N_{S}$)	1600–4000	Numerology index (μ)	3
Slots per subframe ($2^{\mu }$)	8	Slots per frame ($S_{\mathrm{Fr}}$)	80
Slot time ($T_{s}$)	0.125 ms	Subframe time	1 ms
Frame time ($T_{\mathrm{Fr}}$)	10 ms	Channel bandwidth	50 MHz
OFDM grid size ($N_{\mathrm{grid}}^{\mathrm{size}}$)	32	Subcarrier spacing	120 kHz
Waveform sample rate (*B*)	122.88 MHz	Tx power ($P_{\mathrm{Tx}}$)	25 dBm
FFT points ($N_{\mathrm{FFT}}$)	1024	Noise figure (NF)	9 dB
Transport block size (TBS)	9480 bits	Antenna temp. ($T_{\mathrm{Ant}}$)	290 K
Highway length (*s*)	800 m	UAV height ($z_{u}$)	100 m
Maximum speed ($v_{\max}$)	23 m/s	Maximum acceleration ($a_{\max}$)	2.5 m/s^2^
Acceleration step ($\Delta a_{u}$)	1.25 m/s^2^	Angular disp. (Δθ)	10°

**Table 3:** Statistical performance analysis across evaluation scenarios.

Direction	Traffic	Method	SNR [dB]		Throughput [Mbps]			BLER		
			Mean	Std.	$P_{50}$	IQR	$P_{90}$	$P_{50}$	IQR	$P_{90}$
One-way	Low	UAV1 Proposed	5.79	0.93	53.73	7.25	61.70	0.47	0.13	0.56
		UAV2 Predefined	3.75	1.37	46.63	11.50	55.49	0.56	0.18	0.77
		UAV3 Static	4.03	0.58	49.45	5.91	55.72	0.58	0.13	0.69
		UAV4 Centroid	5.65	0.88	54.99	7.25	61.31	0.46	0.17	0.59
	High	UAV1 Proposed	5.46	0.54	54.93	4.96	59.35	0.45	0.10	0.61
		UAV2 Predefined	4.61	0.90	49.68	8.23	56.16	0.52	0.09	0.63
		UAV3 Static	4.01	0.26	50.77	6.15	54.63	0.56	0.12	0.68
		UAV4 Centroid	5.41	0.52	55.36	6.18	59.45	0.47	0.12	0.62
Two-way	Low	UAV1 Proposed	5.48	0.47	53.47	4.08	56.70	0.48	0.09	0.56
		UAV2 Predefined	4.44	1.11	48.81	8.94	53.96	0.53	0.09	0.63
		UAV3 Static	4.15	0.28	49.55	3.57	52.80	0.57	0.09	0.64
		UAV4 Centroid	5.42	0.47	53.75	3.41	56.57	0.49	0.08	0.57
	High	UAV1 Proposed	5.63	0.56	51.66	4.56	55.56	0.45	0.09	0.53
		UAV2 Predefined	4.48	1.12	46.56	9.25	53.83	0.51	0.09	0.62
		UAV3 Static	4.29	0.30	46.80	4.28	50.70	0.56	0.08	0.66
		UAV4 Centroid	5.52	0.50	51.69	5.64	55.64	0.46	0.11	0.56

## Data Availability

Data are available at https://github.com/ividalcar/UAV-Aided-Vehicular-Networks-A-Trajectory-Optimization-Approach-for-5G-Communication-on-Highways (accessed on 5 August 2026).

## Abbreviations

The following abbreviations are used in this manuscript:

2D	two-dimensional
3GPP	3rd Generation Partnership Project
5G	fifth generation
AoI	age of information
BLER	block error rate
BS	base station
CDL	clustered delay line
CRC	cyclic redundancy check
DLSCH	downlink shared channel coding
ECDF	empirical cumulative distribution function
FFT	Fast Fourier Transform
HARQ	hybrid automatic repeat request
IoV	Internet of Vehicles
IQR	interquartile range
LDPC	low density parity check
LOS	line of sight
MAC	medium access control
MCS	modulation and coding scheme
MEC	mobile edge computing
NLOS	non-line of sight
NOMA	non-orthogonal multiple access
OFDM	orthogonal frequency-division multiplexing
PDCP	packet data convergence protocol
PDSCH	physical downlink shared channel
QoS	quality of service
RB	resource block
RE	resource element
RF	radio frequency
RLC	radio link control
RMa	rural macro cell
RRC	radio resource control
RSU	roadside unit
SCS	subcarrier spacing
SINR	signal to interference plus noise ratio
SNR	signal to noise ratio
SUMO	Simulation of Urban MObility
TBS	transport block size
TraCI	Traffic Control Interface
UAV	unmanned aerial vehicle
